# Moderate full-fat and low-fat yoghurt consumption correlates with reduced mortality risk: a large-scale prospective analysis

**DOI:** 10.7189/jogh.15.04014

**Published:** 2025-01-17

**Authors:** Zhengjun Lin, Min Zeng, Zijian Sui, Yanlin Wu, Xianzhe Tang, Tang Liu

**Affiliations:** 1 Department of Orthopedics, The Second Xiangya Hospital, Central South University, Changsha, Hunan, China; 2 Department of Statistics and Finance, School of Management, University of Science and Technology of China, Hefei, China; 3 Department of Orthopedics, Chenzhou No.1 People's Hospital, Chenzhou, Hunan, China; 4 National Clinical Research Center for Metabolic Disease, Department of Metabolism and Endocrinology, The Second Xiangya Hospital, Central South University, Changsha, Hunan, China

## Abstract

**Background:**

Yoghurt is a commonly consumed fermented food recommended by many guidelines. Yoghurt consumption can contribute to the intake of multiple nutrients and reduce the risk of several diseases. However, prospective evidence is limited on the associations between full/low-fat yoghurt consumption and mortality risk. In this prospective cohort study, we aimed to assess the dose-dependent associations between full/low-fat yoghurt intake and all-cause or cause-specific mortality.

**Methods:**

We enrolled 186 168 participants from the UK Biobank who had joined the study between 2006 and 2010 and were followed up until 2022. We obtained data on self-reported intake of full/low-fat yoghurt and mortality from all causes and specific causes of death, including cancers and cardiovascular diseases (CVDs). We then used Cox proportional hazard models to calculate the hazard ratio (HR) and 95% confidence interval (CI) to evaluate the associations between full-fat and low-fat yoghurt intake and mortality. Lastly, we conducted subgroup and sensitivity analyses to examine the robustness of our findings.

**Results:**

A total of 9402 deaths occurred during a mean follow-up of 13.4 years, including 1687 CVD-related and 5073 cancer-related deaths. Relative to non-consumers, the HRs (95% CIs) for all-cause mortality risk in participants consuming >0–50, 50–100, and >100 g of full-fat yoghurt a day were 0.82 (95% CI = 0.72, 0.93), 0.97 (95% CI = 0.86, 1.09), and 0.96 (95% CI = 0.84, 1.1) respectively. The corresponding HR estimates relative to non-consumers for participants consuming low-fat yoghurt were 0.88 (95% CI  = 0.81, 0.95), 0.91 (95% CI = 0.85, 0.98), and 0.95 (95% CI = 0.89, 1.01), respectively. Subgroup analysis indicated women who had moderate consumption of full-fat yoghurt had lower all-cause mortality risk, while men consuming low-fat yoghurt had lower all-cause mortality risk.

**Conclusions:**

Moderate consumption of full-fat and low-fat yoghurt was correlated with decreased all-cause mortality. Future cohort studies are warranted to verify the potential of adopting yoghurt consumption as part of a healthy diet to reduce mortality.

Yoghurt, produced by the bacterial fermentation of milk, is among the most widely consumed fermented foods [1,2]. Due to an increasing focus on the gut microbiome, numerous studies have investigated the effects of exogenous microbes from the diet on health improvement and disease prevention [3,4]. Yoghurt consumption has consequently been confirmed as part of a balanced and healthy dietary strategy globally [5–7], with epidemiological studies suggesting that yoghurt consumption is closely related to reduced risks of several diseases, including type 2 diabetes, cardiovascular disease (CVD), and metabolic syndrome. For instance, it has been reported that the consumption of 80–125 g fo yoghurt a day can reduce the risk of type 2 diabetes by 14% [8].

In clinical practice, the inclusion of yoghurt in dietary recommendations can have significant implications for patient health. The probiotic content in yoghurt can positively impact gut health, improve digestion, and enhance immune function [9,10]. For individuals with gastrointestinal disorders, yoghurt consumption can aid in maintaining a healthy gut microbiota and alleviate symptoms such as bloating and indigestion [1]. Moreover, the calcium and vitamin D content in yoghurt can support bone health and reduce the risk of osteoporosis and bone fracture, particularly in ageing populations [11].

Recently, several studies have explored the associations of yoghurt and other fermented foods containing live bacteria consumption with mortality. A recent study of two USA cohorts has shown that yoghurt consumption was significantly correlated with reduced all-cause and cancer-specific mortality risks in women, although it detected no clear dose-response relationship [12]. However, another cohort study based on 32 625 adults in the National Health and Nutrition Examination Survey (NHANES) found that yoghurt consumption was significantly associated with reduced all-cause mortality risks, although it was not significantly associated with CVD- and cancer-specific mortality [13]. These contradictory and heterogeneous findings suggest that further investigation into the health benefits of yoghurt consumption in a larger cohort study is warranted.

Currently, dietary guidelines have recommended preferential consumption of low-fat dairy as a healthy diet for the prevention of CVD and metabolic diseases because the high saturated fatty acid content of full-fat dairy may increase fasting total and low-density lipoprotein cholesterol concentrations, subsequently increasing the risk of several diseases [14,15]. However, dairy products and dairy fat, despite their drawbacks, also contain beneficial compounds which could impact health outcomes, such as specific amino acids, medium-chain and odd-chain saturated fats, milk fat globule phospholipids, unsaturated and branched-chain fats, natural trans fats, vitamins K1 and K2, calcium, and probiotics. It is thus important to consider that the overall effect of consuming dairy products may not be solely determined by a single factor.

Multiple studies have reported contrasting findings regarding the relationship between full-fat dairy consumption and health outcomes, and there is insufficient evidence supporting the differential protective effects of full-fat or low-fat dairy consumption. For instance, a previous meta-analysis has presented an inverse relationship between total and low-fat dairy, and cheese consumption and the risk of type 2 diabetes [16]. Annalisa et al. [17] have reported that moderate dairy consumption has no detrimental effects on CVD prevention and that these effects are not dependent on the fat content. To date, there has been limited evidence on the associations between yoghurt and mortality stratified by the fat content. Therefore, clarifying the exact effects of full-fat and low-fat yoghurt consumption on health outcomes is urgently needed and is critical for making global policy recommendations.

The UK Biobank, a large-scale prospective cohort study of half a million participants in the UK, offers a unique opportunity to explore the associations of yoghurt consumption and different yoghurt types with health outcomes in diverse settings. We aimed to explore the dose-dependent associations of full-fat and low-fat yoghurt consumption with mortality risk from all causes and specific causes within the large-scale cohort, thereby providing critical evidence for the consumption of different yoghurt types as a healthy diet to reduce mortality risk.

## METHODS

### Study participants

**Figure 1:**
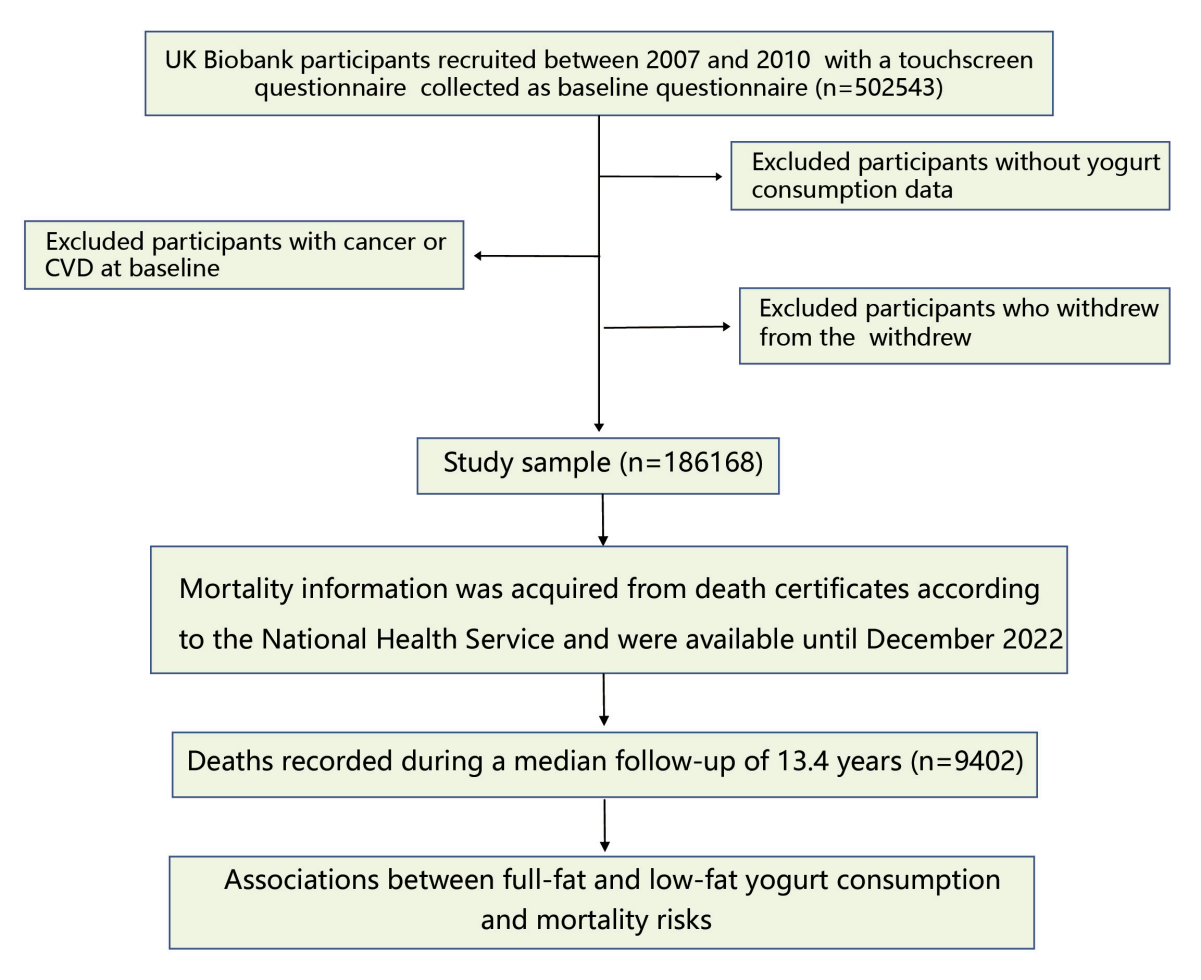
Flowchart of the selection process.

The UK Biobank recruited 502 543 voluntary participants aged 37 to 73 years from the UK between 2007 and 2010 who then completed a touchscreen questionnaire after giving written informed consent, underwent a nurse-led interview, received physical measurements, and provided detailed socio-demographic and lifestyle information, as well as biological samples [18]. For our analysis, we excluded participants who did not provide information about yoghurt consumption (n = 210 950), had a history of cancer or CVD at baseline (n = 186 683), or withdrew from the study (n = 186 168). This left 186 168 participants for further investigation (**Figure 1**).

### Exposure assessment

Yoghurt consumption was evaluated at baseline using a touchscreen questionnaire called the Oxford WebQ, a 24-hour dietary recall questionnaire. Specifically, participants were asked to complete five online questionnaires between April 2009 and June 2012 and were required to report the volume and type of yoghurt (plain or flavoured) consumed during the previous 24 hours each time. Based on these responses, Piernas et al. [19] summarised the full-fat or low-fat yoghurt consumption in the last 24 hours for each participant (field IDs 26096 and 26102, respectively). At the time, major UK supermarkets adhered to EU regulations, which defined low fat as ≤3 g of fat/100 g or ≤1.5 g/100 mL [20]. We further categorised participants into four subgroups: non-consumers, full-fat yoghurt consumers (participants only consumed full-fat yoghurt), low-fat yoghurt consumers (participants only consumed low-fat yoghurt), and participants consuming both yoghurt types. We classified participants as yoghurt consumers if they had consumed yoghurt in at least one of the dietary recalls, and as  non-consumers otherwise. We calculated the mean volume of yoghurt consumed across multiple recalls as the exposure variable for yoghurt consumption.

### Outcomes assessment

We obtained mortality data from death certificates stored by the National Health Service Information Centre (England and Wales) and the National Health Service Central Register Scotland (Scotland) until December 2022. We censored participants in the mortality analysis at this censoring date or the date of death, whichever occurred first. We classified specific causes of death according to the International Classification of Diseases, 10th Revision (ICD-10). Our analysis focussed on all-cause mortality and mortality due to CVD (codes I00 to I99) and cancer (codes C00 to D48).

### Covariates assessment

Baseline questionnaires gathered information on various covariates, including demographic variables (age, sex, ethnicity, Townsend deprivation index (TDI), education level), smoking status, physical activity, body mass index (BMI), medical information (hypertension, diabetes, high cholesterol, long-standing illness, drug use), and dietary intake of various nutrients and substances (including milk and other dairy, coffee, tea, sugar-sweetened beverages (SSBs), artificially sweetened beverages (ASBs), fresh fruit, vegetables, red meat, processed meat, vitamin and mineral supplements, total energy intake and total fat intake).

Among these, BMI was calculated as weight divided by height squared. Negative values of the TDI indicated relative affluence. Long-standing illness was self-reported with a binary response (‘yes’ or ‘no’) by each participant to the question ‘Do you have any long-standing illness, disability, or infirmity?’ to assess the overall health status. Drug use includes cholesterol-lowering drug use, blood pressure drug use, and insulin drug use. The Physical Activity Questionnaire (IPAQ) was used to evaluate physical activity levels. Total energy intake and total fat intake were estimated from participants’ answers to the dietary questionnaire [21]. We used multivariate imputation by chained equations to imput any missing covariate data.

### Statistical analysis

We summarised baseline characteristics as percentages for categorical variables and as means and standard deviations (SDs) for continuous variables across yoghurt intake categories, after which we compared them across groups using χ^2^ tests and analysis of variance (ANOVA). We then used Cox proportional hazard models to assess the prospective associations between full-fat and low-fat yoghurt consumption and mortality risks, presenting the results as hazard ratios (HRs) and 95% confidence intervals (CIs). We also used restricted cubic spline regression to explore the dose-dependent relationship between yoghurt consumption and mortality risks, whereby we categorised yoghurt consumers into three subgroups based on the volume of intake per day. We assessed the proportional hazards assumption using Schoenfeld residuals and found no evidence of serious violation. We conducted all analyses based on the three-model approach categorised by different covariates, where we adjusted model 1 for demographic variables, additionally adjusted model 2 for smoking status, alcohol consumption, BMI, physical activity level, and medical information, and further adjusted model 3 for dietary intake of various nutrients and substances.

We alsoe performed subgroup analyses by age (≥60 years vs. <60 years), sex (male vs. female), TDI (greater than or equal to average vs. less than average), smoking (yes vs. no), alcohol intake (yes vs. no), vitamin intake (yes vs. no), minerals intake (yes vs. no), BMI (≥30 kg/m2 vs. <30 kg/m2), hypertension (yes vs. no), diabetes (yes vs. no), and physical activity (high vs. low). We also conducted further sensitivity analyses were to validate the primary findings, where we excluded participants with outcome events during the first two years of follow-up, those with missing covariates, those with vitamin or mineral intake, those who reported less than two measurements of yoghurt consumption, and those who exhibited high within-person variability (standard difference greater than 50 g per day) across the five assessments. We further adjusted for certain environmental factors, including particulate matter (PM) intake (PM_2.5_, PM_2.5–10_, PM_10_), NOx, average sound level of noise pollution, inverse distance to major roads, and green space percentage.

We performed all analyses in R, version 4.1.1 (R Core Team, Vienna, Austria). A *P*-value <0.05 indicated statistical significance.

## RESULTS

### Baseline characteristics

Among the 186 168 participants, 86 272 (46.34%) were yoghurt consumers. Low-fat yoghurt (29.15%) was the most commonly consumed yoghurt type, followed by full-fat yoghurt (6.98%) and both types (10.20%). Non-consumers of yoghurt were more likely to report diabetes, high cholesterol, and hypertension, and they had the highest alcohol consumption frequencies. Consumers of full-fat yoghurt had the lowest BMI and the lowest rates of diabetes and hypertension. Consumers of low-fat yoghurt and both yoghurt types had a higher intake of vegetables and fruit and were more likely to be from a higher social class (**Table 1**).

**Table 1:** Demographic and lifestyle characteristics of participants in the UK Biobank study*

Characteristics	Total	Non-consumers	Low-fat yoghurt	Full-fat yoghurt		Both		*P*-value
Participants	186 168	99 896	54 272		13 003		18 997	
Age in years, x̄ (SD)	55.58 (7.95)	55.05 (8.07)	56.25 (7.70)		55.15 (8.06)		56.78 (7.61)	<0.001
Male sex	82 863 (44.5)	50 364 (50.4)	19 986 (36.8)		5876 (45.2)		6637 (34.9)	<0.001
TDI, x̄ (SD)	−1.58 (2.87)	−1.44 (2.94)	−1.80 (2.74)		−1.48 (2.90)		−1.73 (2.76)	<0.001
White ethnicity	168 914 (90.8)	90 162 (90.3)	49 942 (92.1)		11 483 (88.4)		17327 (91.2)	<0.001
College or university degree education	80 352 (43.2)	41 436 (41.5)	22 332 (41.2)		7378 (56.8)		9206 (48.5)	<0.001
Physical time, x̄ (SD)	2492.12 (2320.06)	2468.24 (2364.34)	2525.31 (2273.45)		2478.29 (2278.38)		2532.36 (2242.22)	<0.001
BMI, x̄ (SD)	26.88 (4.58)	27.10 (4.65)	26.96 (4.55)		25.73 (4.21)		26.27 (4.37)	<0.001
Hypertension	30 264 (16.9)	17 228 (17.9)	8318 (15.9)		1894 (15.2)		2824 (15.5)	<0.001
Diabetes	6855 (3.7)	4288 (4.3)	1814 (3.3)		250 (1.9)		503 (2.7)	<0.001
High cholesterol	20 948 (11.3)	11 761 (11.8)	6238 (11.5)		1001 (7.7)		1948 (10.3)	<0.001
Smoking status								<0.001
*Never*	106 957 (57.6)	55 439 (55.7)	32 259 (59.6)		7834 (60.4)		11425 (60.3)	
*Sometimes*	64 054 (34.5)	34 313 (34.4)	18 922 (34.9)		4283 (33.0)		6536 (34.5)	
*Often*	14 669 (7.9)	9867 (9.9)	2961 (5.5)		851 (6.6)		990 (5.2)	
Alcohol in g/d, x̄ (SD)	16.25 (21.23)	18.33 (23.59)	13.49 (17.89)		15.48 (18.61)		13.78 (17.04)	<0.001
Long-standing illness	49 155 (26.9)	27 183 (27.8)	14 139 (26.6)		3061 (24.0)		4772 (25.6)	<0.001
Cholesterol-lowering drug use	22 635 (12.2)	12 842 (12.9)	6686 (12.4)		1028 (7.9)		2079 (11.0)	<0.001
Blood pressure drug use	29 302 (15.8)	16 465 (16.6)	8665 (16.0)		1454 (11.2)		2718 (14.4)	<0.001
Insulin drug use	1374 (0.7)	831 (0.8)	377 (0.7)		54 (0.4)		112 (0.6)	<0.001
Total energy intake in KJ/d, x̄ (SD)	8853.61 (2728.31)	8840.68 (2866.36)	8719.45 (2535.98)		9435.54 (2689.07)		8906.58 (2476.75)	<0.001
Total fat intake in g/d (SD)	73.34 (0.83)	73.35 (0.83)	73.34 (0.83)		73.36 (0.83)		73.35 (0.83)	0.019
Yoghurt intake in g/d, x̄ (SD)	43.37 (59.43)	0.00 (0.00)	95.30 (55.99)		79.14 (49.51)		98.59 (49.59)	<0.001
Low-fat yoghurt intake in g/d, x̄ (SD)	9.73 (27.66)	0.00 (0.00)	0.00 (0.00)		79.14 (49.51)		41.21 (27.62)	<0.001
Full-fat yoghurt intake in g/d, x̄ (SD)	33.64 (53.92)	0.00 (0.00)	95.30 (55.99)		0.00 (0.00)		57.38 (37.21)	<0.001
Vegetables in servings/d, x̄ (SD)	4.89 (3.28)	4.71 (3.25)	5.16 (3.30)		4.91 (3.30)		5.08 (3.26)	<0.001
Fruit in servings/d, x̄ (SD)	3.09 (2.55)	2.77 (2.51)	3.51 (2.59)		3.19 (2.37)		3.53 (2.46)	<0.001
Process meat in servings/d, x̄ (SD)	1.82 (1.07)	1.91 (1.07)	1.70 (1.03)		1.78 (1.08)		1.68 (1.05)	<0.001
Red meat in servings/d, x̄ (SD)	3.60 (1.76)	3.69 (1.77)	3.50 (1.71)		3.56 (1.82)		3.47 (1.77)	<0.001
Water intake in drinks/d, x̄ (SD)	2.71 (2.28)	2.63 (2.31)	2.83 (2.26)		2.71 (2.23)		2.78 (2.21)	<0.001
Coffee consumption	142 604 (76.6)	73 980 (74.1)	42 952 (79.1)		10 292 (79.2)		15 380 (81.0)	<0.001
Tea consumption	155 482 (83.5)	81 086 (81.2)	46 549 (85.8)		11 273 (86.7)		16 574 (87.2)	<0.001
Milk pudding	14 753 (7.9)	7342 (7.3)	4501 (8.3)		1105 (8.5)		1805 (9.5)	<0.001
Milk chocolate	19 766 (10.6)	8340 (8.3)	6408 (11.8)		2052 (15.8)		2966 (15.6)	<0.001
Cheese	113 670 (61.1)	55 779 (55.8)	34 780 (64.1)		9377 (72.1)		13 734 (72.3)	<0.001
Milk	178 040 (95.7)	94 688 (94.9)	52 507 (96.8)		12 464 (95.9)		18 381 (96.8)	<0.001
Vitamin	28 147 (15.1)	13 965 (14.0)	8931 (16.5)		2082 (16.0)		3169 (16.7)	<0.001
Minerals	43 062 (23.1)	21 416 (21.4)	13 887 (25.6)		2954 (22.7)		4805 (25.3)	<0.001
SSBs	66 119 (35.5)	34 006 (34.0)	19 427 (35.8)		4945 (38.0)		7741 (40.7)	<0.001
ASBs	38 562 (20.7)	19 204 (19.2)	13 453 (24.8)		1717 (13.2)		4188 (22.0)	<0.001

ASBs – artificially sweetened beverages, BMI – body mass index, CVD – cardiovascular disease, SSBs – sugar-sweetened beverages, TDI – townsend deprivation index

*Presented as n (%) unsless specified otherwise.

### Yoghurt consumption and mortality risk

**Figure 2:**
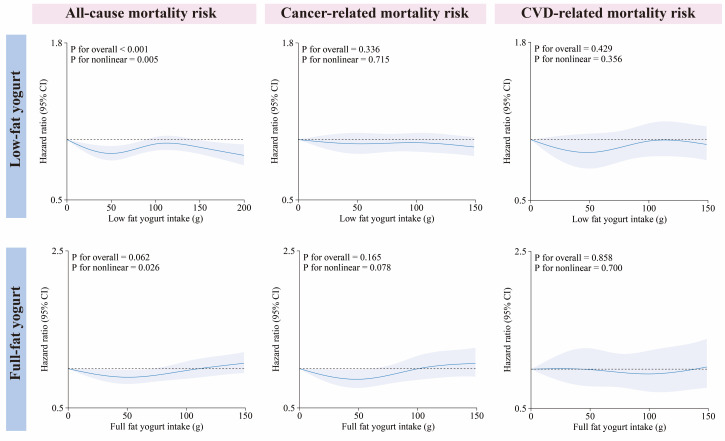
Dose-response associations of full-fat and low-fat yoghurt with all-cause, cancer, and CVD mortality.

During a mean follow-up of 13.4 years, we observed 9402 deaths, of which 5073 were cancer-related and 1687 CVD-related. Initially, we evaluated the associations of types of yoghurt intake with mortality risks. In unadjusted Cox models (model 0), there were inverse associations between low-fat yoghurt and full-fat yoghurt consumption and all-cause, CVD-related, and cancer-related mortality risks, as compared to non-consumption (Table S1 in the **Online Supplementary Document**). After multivariable adjustment for confounding factors (model 3), a marginally significant association remained specifically for all-cause mortality and low-fat yoghurt consumption (HR = 0.93; 95% CI = 0.88, 0.97) or full-fat yoghurt consumption (HR = 0.91; 95% CI = 0.83, 0.99). We additionally investigated the dose-dependence between yoghurt intake and mortality risks, and we found a nonlinear associations between low-fat yoghurt and full-fat yoghurt consumption and all-cause mortality risk (**Figure 2**). However, the associations between low-fat and full-fat yoghurt intake with CVD-related and cancer-related mortality risk were not statistically significant because of the wide 95% CIs.

We further divided low-fat and full-fat yoghurt intake into three categories: 0–50, 50–100, and >100 g/d. In unadjusted Cox models (model 0), the consumption of low-fat and full-fat yoghurt was significantly correlated with lower all-cause mortality risk in all categories, and there were significant associations between the consumption of low-fat (0–50 g/d) and full-fat (0–50 g/d) yoghurt with cause-specific mortality risk (**Table 2**). After multivariable adjustment (model 3), the HRs for low-fat yoghurt intake of 0–50, 50–100, and >100 g/d were 0.88 (95% CI = 0.81, 0.95), 0.91 (95% CI = 0.85,0.98), and 0.95 (95% CI = 0.89–1.01) respectively, when compared to non-yoghurt consumption and all-cause mortality. The HRs for full-fat yoghurt intake of 0–50, 50–100, and >100 g/d were 0.82 (95% CI = 0.72, 0.93), 0.97 (95% CI = 0.86, 1.09), and 0.96 (95% CI = 0.84, 1.1) respectively. However, we observed no statistically significant differences between the low-fat and full-fat yoghurt intake and cause-specific mortality risks in both causes. This suggests that moderate consumption of low-fat yoghurt (<100g/d) and consumption of full-fat yoghurt (<50g/d) were correlated with reduced all-cause mortality risk.

**Table 2:** Dose-response associations of low-fat and full-fat yoghurt consumption with all-cause, cancer, and CVD mortality*

		Yoghurt consumers, HR (95% CI)		
	**Non-consumers**	**<50 g/d**	**50–100 g/d**	**>100 g/d**
**Low-fat yoghurt**				
All-cause				
*Events, n (%)*	5391 (5.40)	943 (4.34)	1177 (4.77)	1338(4.98)
*Model 0*	1 (ref)	0.79 (0.74, 0.86)	0.86 (0.80, 0.92)	0.92 (0.86, 0.97)
*Model 1*	1 (ref)	0.81 (0.75, 0.87)	0.84 (0.79, 0.90)	0.90 (0.85, 0.96)
*Model 2*	1 (ref)	0.85 (0.78, 0.92)	0.88 (0.83-0.95)	0.94 (0.88, 0.99)
*Model 3*	1 (ref)	0.88 (0.81, 0.95)	0.91 (0.85, 0.98)	0.95 (0.89, 1.01)
Cancer				
*Events, n (%)*	2838 (2.84)	544 (2.50)	660 (2.68)	717 (2.67)
*Model 0*	1 (ref)	0.88 (0.79, 0.97)	0.94 (0.86, 1.03)	0.93 (0.86, 1.01)
*Model 1*	1 (ref)	0.87 (0.78, 0.96)	0.90 (0.82, 0.98)	0.89 (0.82, 0.97)
*Model 2*	1 (ref)	0.91 (0.82, 1.01)	0.94 (0.86, 1.03)	0.92 (0.85, 1.00)
*Model 3*	1 (ref)	0.93 (0.84, 1.04)	0.97(0.88, 1.06)	0.95 (0.87, 1.03)
CVD				
*Events, n (%)*	981 (0.98)	163 (0.75)	211 (0.86)	240 (0.89)
*Model 0*	1 (ref)	0.80 (0.66, 0.96)	0.86 (0.73, 1.01)	0.91 (0.79, 1.05)
*Model 1*	1 (ref)	0.83 (0.69, 1.00)	0.87 (0.74, 1.02)	0.94 (0.82, 1.09)
*Model 2*	1 (ref)	0.89 (0.74, 1.07)	0.92 (0.78, 1.09)	0.98 (0.85, 1.14)
*Model 3*	1 (ref)	0.93 (0.77, 1.12)	0.95 (0.81, 1.13)	0.99 (0.85, 1.14)
**Full-fat yoghurt**				
All-cause				
*Events, n (%)*	5391 (5.40)	740 (4.23)	462 (4.86)	241(4.80)
*Model 0*	1 (ref)	0.68 (0.60, 0.77)	0.80 (0.71, 0.90)	0.86 (0.75, 0.97)
*Model 1*	1 (ref)	0.71 (0.63, 0.80)	0.85 (0.76, 0.96)	0.88 (0.77, 1.00)
*Model 2*	1 (ref)	0.78 (0.69, 0.88)	0.93 (0.83, 1.05)	0.96 (0.84, 1.09)
*Model 3*	1 (ref)	0.82 (0.72, 0.93)	0.97 (0.86, 1.09)	0.96 (0.84, 1.10)
Cancer				
*Events, n (%)*	2838 (2.84)	426 (2.44)	240 (2.52)	137 (2.73)
*Model 0*	1 (ref)	0.77 (0.65, 0.90)	0.82 (0.70, 0.96)	0.93 (0.78, 1.10)
*Model 1*	1 (ref)	0.79 (0.67, 0.92)	0.86 (0.73, 1.01)	0.94 (0.79, 1.12)
*Model 2*	1 (ref)	0.85 (0.72, 1.00)	0.92 (0.79, 1.09)	1.01 (0.85, 1.21)
*Model 3*	1 (ref)	0.88 (0.75, 1.04)	0.94 (0.80, 1.11)	1.02 (0.85, 1.21)
CVD				
*Events, n (%)*	981 (0.98)	118 (0.68)	84 (0.88)	38 (0.76)
*Model 0*	1 (ref)	0.59 (0.44, 0.79)	0.79 (0.59, 1.05)	0.75 (0.54, 1.04)
*Model 1*	1 (ref)	0.62 (0.46, 0.84)	0.86 (0.65, 1.14)	0.78 (0.56, 1.08)
*Model 2*	1 (ref)	0.73 (0.54, 0.99)	1.00 (0.75, 1.33)	0.89 (0.64, 1.24)
*Model 3*	1 (ref)	0.77 (0.57, 1.05)	1.03 (0.78, 1.38)	0.87 (0.63-1.22)

CI – confidence interval, CVD – cardiovascular disease, HR – hazard ratio, ref – reference

*Model 0: unadjusted. Model 1: adjusted for sex, age, TDI, ethnicity and education. Model 2: further adjusted from model 1 for smoking status, BMI, physical activity level, alcohol consumption, high cholesterol, hypertension, diabetes, long-standing illness, cholesterol-lowering drug use, blood pressure drug use, insulin drug use. Model 3: further adjusted from models 1 and 2 for vitamin and mineral supplement use (yes or no (vitamin A, B, C, D, or E; folic acid; or multivitamins or minerals)), tea consumption, SSBs, ASBs, and intake of total energy, fat, total sugar, fresh fruit, vegetables, red meat, processed meat, water, coffee, milk pudding, milk chocolate, cheese, and milk.

### Subgroup and sensitivity analysis

**Figure 3:**
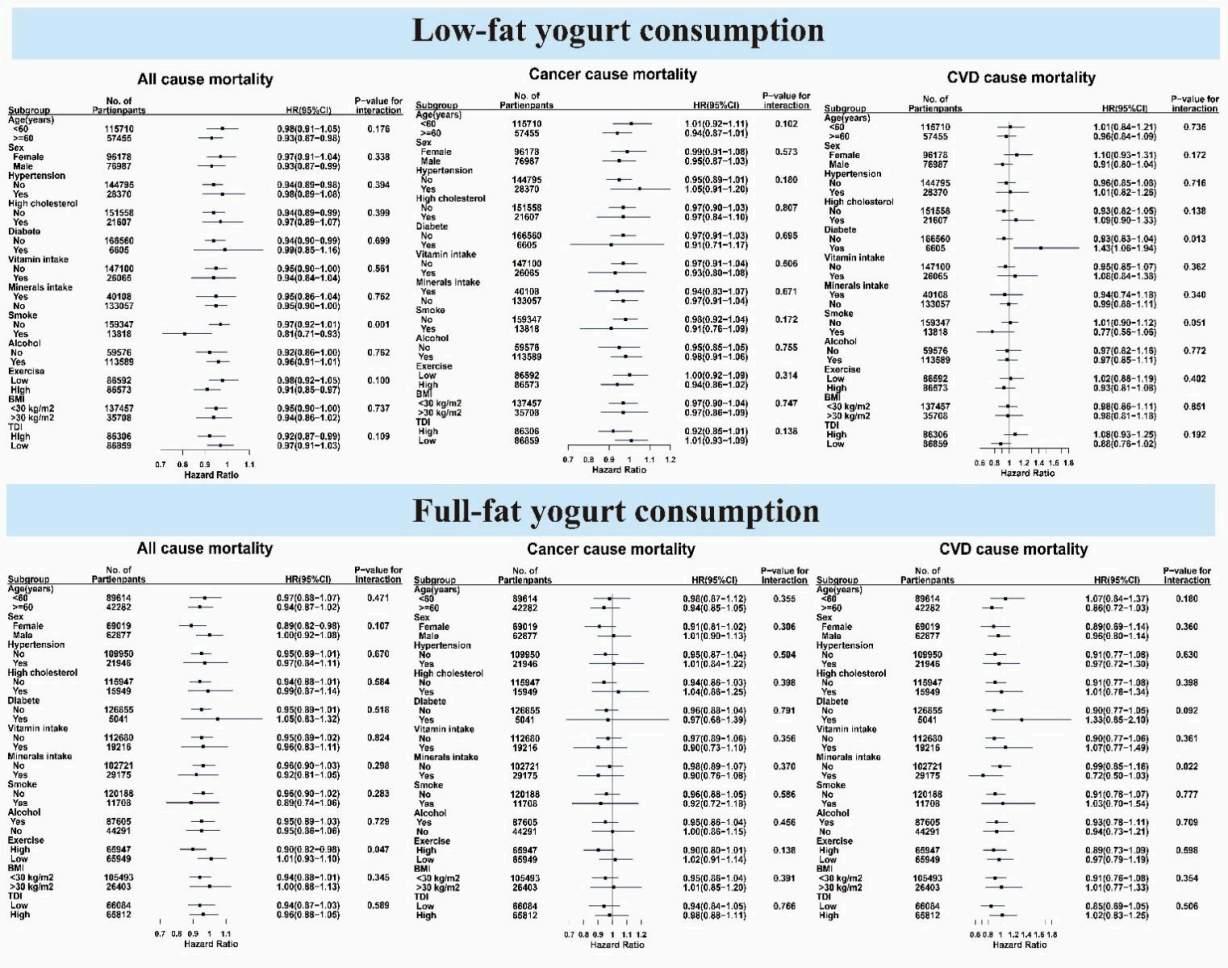
Associations of full-fat and low-fat yoghurt with all-cause, cancer, and CVD mortality stratified by potential factors.

We performed subgroup analyses to evaluate the correlation between low-fat and full-fat yoghurt intake and mortality risks in different subgroups (**Figure 3**). Compared with non-consumption, the consumption of full-fat yoghurt was associated with decreased all-cause mortality in female participants (HR = 0.89; 95% CI = 0.82, 0.98), and those with high physical activity (HR = 0.9; 95% CI = 0.82, 0.98), while the intake of low-fat yoghurt was correlated with decreased mortality risks from all causes in males (HR = 0.93; 95% CI = 0.87, 0.99), as well as participants older than 60 years (HR = 0.93; 95% CI = 0.87, 0.98), without hypertension (HR = 0.94; 95% CI = 0.89, 0.98), without high cholesterol (HR = 0.94; 95% CI = 0.89, 0.99), without diabetes (HR = 0.94; 95% CI = 0.9, 0.99), with high physical activity (HR = 0.91; 95% CI = 0.85, 0.97), high TDI values (HR = 0.92; 95% CI = 0.87, 0.99), and those who are smokers (HR = 0.81; 95% CI = 0.71, 0.93). The findings remained generally consistent in our sensitivity analyses (Tables S2–7 in the **Online Supplementary Document**).

## DISCUSSION

The findings of our large-scale prospective study showed that the consumption of full-fat and low-fat yoghurt was associated with reductions in risk for all-cause mortality. Dose-dependent investigation indicated that consumption of full-fat yoghurt (<50 g/d) and low-fat yoghurt (<100 g/d) were correlated with reduced all-cause mortality, and that such effects may be attenuated by excessive yoghurt intake. We found no significant associations between higher full-fat and low-fat yoghurt consumption and CVD-specific and cancer-specific mortality risk. These findings support the assumption that moderate intake of full-fat and low-fat yoghurt in the diet may effective for reducing the risk of mortality.

Yoghurt is a nutritious fermented dairy product that has been widely recognised as a healthy diet in most countries [22]. Aside from containing probiotic strains, it is also a good source of rich micronutrients and macronutrients, such as calcium, zinc, vitamins, and peptides, which are essential for maintaining body function [1]. There is growing epidemiological and clinical evidence that yoghurt consumption may be beneficial for weight regulation and metabolic risk factors. However, the impact of dairy products and their components on cardiovascular risk and mortality risks is still being discussed. Dairy products are a major source of saturated fat, which is thought to affect blood lipids and increase CVDs and mortality. Under this framework, dietary guidelines recommend minimising the consumption of full-fat dairy products and prioritising low-fat products to prevent CVDs in the population, although the evidence for this is sparse and inconsistent. For instance, a previous dose-response meta-analysis of prospective cohort studies has found no associations between total intake of dairy (including high-fat/low-fat) and the mortality risk, coronary heart disease, or CVD [23]. An investigation of three prospective cohort studies also saw no inverse association between high intake of total dairy and mortality risk, and it linked the consumption of whole milk to increasing all-cause mortality risk (HR per 0.5 additional serving/d = 1.11, 95% CI = 1.09, 1.14), CVD-related mortality (HR = 1.09; 95% CI = 1.03, 1.15), and cancer-related mortality (HR = 1.11; 95% CI = 1.06, 1.17) [24]. A prospective study investigated the relationship between the intake of full-fat milk (HR = 1.20; 95% CI = 0.99, 1.44) or low-fat milk (HR = 0.88; 95% CI = 0.80, 0.96) with all-cause mortality risk and found that high consumption of low-fat milk may reduce mortality risk. In contrast, prior research found that full-fat milk may be related to higher all-cause mortality [25]. A cohort study from the US National Health and Nutrition Examination Survey 1999–2014 showed that the consumption of low-fat or skim milk was significantly related to reduced all-cause and CVD-related mortality risk compared with whole milk, indicating that low-fat milk may be more beneficial for the prevention of CVD in adults [26]. These contrasting findings may be attributed to sample heterogeneity, and they suggest that the potential of full-fat and low-fat dairy consumption in a healthy diet to reduce mortality risk warrants further investigation.

Few studies have sought to distinguish the full-fat and low-fat yoghurt consumption in the diet on disease and mortality risks. Our findings indicate that the moderate consumption of low-fat yoghurt (<100 g/d) and full-fat yoghurt (<50 g/d) were correlated with reduced all-cause mortality risk. Previous research has explored the associations of yoghurt intake with mortality risks and reported contrasting results. One meta-analysis indicated that the consumption of total fermented dairy (including sour milk products, yoghurt, and cheese, per 20 g/d) was negatively associated with mortality risks and CVD risk; however, yoghurt consumption (three populations) was not significantly associated with all-cause mortality (risk ratio (RR) = 0.97; 95% CI = 0.85, 1.11; *I*^2^ = 65.8%; *P* = 0.054) [23]. Another study reported a contradictory finding where, compared with the lowest quintile, the highest yoghurt intake was related to lower mortality risk from all causes (HR = 0.89; 95% CI = 0.79, 1.0), a lower CVD-related mortality risk (HR = 0.84; 95% CI = 0.70, 1.0), and a lower coronary heart disease-related mortality risk (HR = 0.73; 95% CI = 0.57, 0.9), but not to mortality from total cancer, gastrointestinal cancer, or other cancers [27].

The potential mechanisms underlying the health promotion effects of yoghurt consumption are complex. Yoghurt is a rich source of essential nutrients such as protein, vitamins (B12, riboflavin), and minerals (calcium and phosphorus) [28,29]. These nutrients contribute to overall health and support various body functions. Additionally, yoghurt is a critical source of health-promoting bacteria and can help improve intestinal and extraintestinal health [22,30]. It has been found that dietary yoghurt intake can maintain systemic glucose homeostasis and prevent liver insulin resistance and liver steatosis [31]. Fecal microbiota transplantation experiments further confirmed that these effects are partly related to the gut microbiota. Hence, yoghurt intake may influence the gut microbiota and intestinal health to prevent premature death [32]. Full-fat dairy products have a high percentage of C-12 to C-18 fatty acids in dairy fat, which are proposed to have a negative influence on health [33]. The relationship between saturated fat acid intake and health also depends on the type of fatty acids specific to foods or other nutrients. Other nutrients in foods may also play a role in modulating some of the negative properties of certain saturated fats [14,24]. Additional research and clinical trials are required to better understand the biological mechanisms between full-fat and low-fat yoghurt consumption and health outcomes.

Here we observed no significant association between the consumption of full-fat and low-fat yoghurt and the risk of CVD-related and cancer-related death. These findings are consistent with several previous publications. Cao et al. [34] also reported no association between yoghurt consumption and mortality risk due to CVDs (HR = 0.92; 95% CI = 0.81, 1.03) and cancer (HR = 0.97; 95% CI = 0.83, 1.12). A previous study also suggests that yoghurt intake has no association with cancer-related mortality risk [35]. However, a two-cohort study in the USA suggested that, compared with low consumption, high yoghurt consumption was associated with reduced cancer mortality risk in women (HR = 0.87; 95% CI = 0.78, 0.98; *P*-value for trend = 0.04) [36]. A community-based cohort study in Japan also indicated that the consumption of yoghurt and milk intake was independently associated with reduced all-cause and cancer-related mortality risks [37]. The association between yoghurt consumption and cancer-related mortality may depend on the cancer type and characteristics. For instance, full-fat milk consumption was previously found to be associated with a higher risk of cancer mortality, including ovarian cancer and prostate cancer mortality, while the intake of low-fat milk was associated with a lower risk of colorectal cancer mortality [24]. Overall, the effects of yoghurt consumption on mortality from specific causes need to be further explored in future studies.

In our subgroup analyses, we detected the inverse association between full-fat yoghurt consumption and all-cause mortality risk in the female subgroup. In the male subgroup, we found an association between low-fat yoghurt consumption and reduced all-cause mortality risk. We further observed inverse association between low-fat yoghurt intake and total mortality risk in participants older than 60 years old, suggesting that low-fat yoghurt may be a healthy diet choice in older populations. These findings indicate that the intake of dietary fat may exert different effects on health outcomes between different populations, which requires further in-depth investigation.

### Strengths and limitations

Our study has several strengths that contribute to its reliability and significance. We conducted our research using a large sample size of adults from a nationwide prospective cohort, allowing for a comprehensive analysis of the association between yoghurt consumption and mortality risk and therefore providing novel epidemiological evidence in this area. The extended follow-up, large outcome events, and the intensive information on different potential confounders help us to reduce measurement error and biased estimates. However, several potential limitations should also be considered. First, due to the study’s observational design, we cannot exclude the possibility of residual confounding. Despite our model adjusting for several potential confounders, there may still be unobservable factors that could influence the results, such as lifestyle factors that may correlate with low-fat vs. full-fat dairy choices and thus confound the relationship between yoghurt consumption and mortality. This complicates the interpretation of our findings, as it highlights the possibility that healthier lifestyles may contribute to both higher yoghurt consumption and lower mortality risk. Second, the misclassification of yoghurt consumption may influence the reliability of our findings, as estimating the volume of yoghurt consumption may be prone to errors. In addition, the dietary habits of each participant may change over time, and participants may switch from full-fat yoghurt to low-fat yoghurt. Self-reported yoghurt consumption at baseline also may not reflect long-term yoghurt consumption. Measurement error in dietary consumption may be a significant limitation in epidemiological studies, potentially hindering the confirmation of the relationship between dietary intake and health outcomes. While dietary assessment methods are being improved to reduce measurement errors (*e.g.* collecting multiple 24-hour recall surveys), measurement errors are unlikely to be eliminated from dietary assessments. Third, changes in other contents of yoghurt may also significantly influence the validity of these findings. For instance, the addition of sugar to yoghurt was more common in the past and is mostly not being done today. Such variations in yoghurt content could confound the effects on mortality risk and should thus be considered in future research. Besides, although we were able to separate yoghurt by low-fat and full-fat, we could not distinguish between different types of yoghurt given the variety of products available, such as those containing prebiotics, probiotics, sugar, and so on. Further more, our study is based on the UK Biobank cohort, which may not fully represent the general population of the UK, as its participants generally live in less socioeconomically deprived regions, potentially introducing a ‘healthy volunteer’ selection bias. Last, only a few deaths from specific causes were detected due to a short follow-up time, and the relationship between yoghurt intake and certain causes of mortality may not be statistically significant.

## CONCLUSIONS

This prospective study showed that the moderate consumption of full-fat and low-fat yoghurt was associated with reductions in the risk for all-cause mortality. A dose-response analysis suggested that 0–50 g per day of full-fat yoghurt consumption and 0–100 g per day of low-fat yoghurt consumption were associated with reduced all-cause mortality risk, whereas such protective effects were impaired for excess intake. These novel findings hold clinical and public health importance by providing evidence for moderate yoghurt consumption as an element of a healthy diet in maintaining overall health.

## Additional material


Online Supplementary Document

## References

[1] Savaiano DA Hutkins RW Yogurt, cultured fermented milk, and health: a systematic review. Nutr Rev. 2021;79:599–614. 10.1093/nutrit/nuaa013 32447398 PMC8579104

[2] Yanni AE Kartsioti K Karathanos VT The role of yoghurt consumption in the management of type II diabetes. Food Funct. 2020;11:10306–16. 10.1039/D0FO02297G 33211046

[3] Richards EM Li J Stevens BR Pepine CJ Raizada MK Gut Microbiome and Neuroinflammation in Hypertension. Circ Res. 2022;130:401–17. 10.1161/CIRCRESAHA.121.319816 35113664 PMC8852773

[4] Valles-Colomer M Menni C Berry SE Valdes AM Spector TD Segata N Cardiometabolic health, diet and the gut microbiome: a meta-omics perspective. Nat Med. 2023;29:551–61. 10.1038/s41591-023-02260-4 36932240 PMC11258867

[5] Laird E Molloy AM McNulty H Ward M McCarroll K Hoey L Greater yogurt consumption is associated with increased bone mineral density and physical function in older adults. Osteoporos Int. 2017;28:2409–19. 10.1007/s00198-017-4049-5 28462469

[6] El-Abbadi NH Dao MC Meydani SN Yogurt: role in healthy and active aging. Am J Clin Nutr. 2014;99:1263S–70S. 10.3945/ajcn.113.073957 24695886 PMC6410895

[7] Fernandez MA Panahi S Daniel N Tremblay A Marette A Yogurt and Cardiometabolic Diseases: A Critical Review of Potential Mechanisms. Adv Nutr. 2017;8:812–29. 10.3945/an.116.013946 29141967 PMC5682997

[8] Salas-Salvadó J Guasch-Ferré M Díaz-López A Babio N Yogurt and Diabetes: Overview of Recent Observational Studies. J Nutr. 2017;147:1452S–61S. 10.3945/jn.117.248229 28615384

[9] Ashraf R Shah NP Immune system stimulation by probiotic microorganisms. Crit Rev Food Sci Nutr. 2014;54:938–56. 10.1080/10408398.2011.619671 24499072

[10] Zhu L Ying N Hao L Fu A Ding Q Cao F Probiotic yogurt regulates gut microbiota homeostasis and alleviates hepatic steatosis and liver injury induced by high-fat diet in golden hamsters. Food Sci Nutr. 2024;12:2488–501. 10.1002/fsn3.3930 38628190 PMC11016441

[11] Iuliano S Poon S Robbins J Bui M Wang X De Groot L Effect of dietary sources of calcium and protein on hip fractures and falls in older adults in residential care: cluster randomised controlled trial. BMJ. 2021;375:n2364. 10.1136/bmj.n2364 34670754 PMC8527562

[12] Schmid D Song M Zhang X Willett WC Vaidya R Giovannucci EL Yogurt consumption in relation to mortality from cardiovascular disease, cancer, and all causes: a prospective investigation in 2 cohorts of US women and men. Am J Clin Nutr. 2020;111:689–97. 10.1093/ajcn/nqz345 31968071 PMC7049530

[13] Lin P Gui X Liang Z Wang T Association of Yogurt and Dietary Supplements Containing Probiotic Consumption With All-Cause and Cause-Specific Mortality in US Adults: A Population-Based Cohort Study. Front Nutr. 2022;9:803076. 10.3389/fnut.2022.803076 35198588 PMC8858963

[14] Naghshi S Sadeghi O Larijani B Esmaillzadeh A High vs. low-fat dairy and milk differently affects the risk of all-cause, CVD, and cancer death: A systematic review and dose-response meta-analysis of prospective cohort studies. Crit Rev Food Sci Nutr. 2022;62:3598–612. 10.1080/10408398.2020.1867500 33397132

[15] Riccardi G Giosuè A Calabrese I Vaccaro O Dietary recommendations for prevention of atherosclerosis. Cardiovasc Res. 2022;118:1188–204. 10.1093/cvr/cvab173 34229346

[16] Aune D Norat T Romundstad P Vatten LJ Dairy products and the risk of type 2 diabetes: a systematic review and dose-response meta-analysis of cohort studies. Am J Clin Nutr. 2013;98:1066–83. 10.3945/ajcn.113.059030 23945722

[17] Giosuè A Calabrese I Vitale M Riccardi G Vaccaro O Consumption of Dairy Foods and Cardiovascular Disease: A Systematic Review. Nutrients. 2022;14:831. 10.3390/nu14040831 35215479 PMC8875110

[18] Palmer LJ UK Biobank: bank on it. Lancet. 2007;369:1980-2. 10.1016/S0140-6736(07)60924-6 17574079

[19] Piernas C Perez-Cornago A Gao M Young H Pollard Z Mulligan A Describing a new food group classification system for UK biobank: analysis of food groups and sources of macro- and micronutrients in 208,200 participants. Eur J Nutr. 2021;60:2879–90. 10.1007/s00394-021-02535-x 33768317 PMC8275520

[20] Moore JB Horti A Fielding BA Evaluation of the nutrient content of yogurts: a comprehensive survey of yogurt products in the major UK supermarkets. BMJ Open. 2018;8:e021387. 10.1136/bmjopen-2017-021387 30228100 PMC6144340

[21] Perez-Cornago A Pollard Z Young H van Uden M Andrews C Piernas C Description of the updated nutrition calculation of the Oxford WebQ questionnaire and comparison with the previous version among 207,144 participants in UK Biobank. Eur J Nutr. 2021;60:4019–30. 10.1007/s00394-021-02558-4 33956230 PMC8437868

[22] Kok CR Hutkins R Yogurt and other fermented foods as sources of health-promoting bacteria. Nutr Rev. 2018;76:4–15. 10.1093/nutrit/nuy056 30452699

[23] Guo J Astrup A Lovegrove JA Gijsbers L Givens DI Soedamah-Muthu SS Milk and dairy consumption and risk of cardiovascular diseases and all-cause mortality: dose-response meta-analysis of prospective cohort studies. Eur J Epidemiol. 2017;32:269–87. 10.1007/s10654-017-0243-1 28374228 PMC5437143

[24] Ding M Li J Qi L Ellervik C Zhang X Manson JE Associations of dairy intake with risk of mortality in women and men: three prospective cohort studies. BMJ. 2019;367:l6204. 10.1136/bmj.l6204 31776125 PMC6880246

[25] Ma L Hu Y Alperet DJ Liu G Malik V Manson JE Beverage consumption and mortality among adults with type 2 diabetes: prospective cohort study. BMJ. 2023;381:e073406. 10.1136/bmj-2022-073406 37076174 PMC10114037

[26] Wang S Liu Y Cai H Li Y Zhang X Liu J Decreased risk of all-cause and heart-specific mortality is associated with low-fat or skimmed milk consumption compared with whole milk intake: A cohort study. Clin Nutr. 2021;40:5568–75. 10.1016/j.clnu.2021.09.012 34656953

[27] Farvid MS Malekshah AF Pourshams A Poustchi H Sepanlou SG Sharafkhah M Dairy Food Intake and All-Cause, Cardiovascular Disease, and Cancer Mortality: The Golestan Cohort Study. Am J Epidemiol. 2017;185:697–711. 10.1093/aje/kww139 28369205 PMC5860026

[28] Marette A Picard-Deland E Yogurt consumption and impact on health: focus on children and cardiometabolic risk. Am J Clin Nutr. 2014;99:1243S–7S. 10.3945/ajcn.113.073379 24646821

[29] Shiby VK Mishra HN Fermented milks and milk products as functional foods–a review. Crit Rev Food Sci Nutr. 2013;53:482–96. 10.1080/10408398.2010.547398 23391015

[30] Pei R Martin DA DiMarco DM Bolling BW Evidence for the effects of yogurt on gut health and obesity. Crit Rev Food Sci Nutr. 2017;57:1569–83. 10.1080/10408398.2014.883356 25875150

[31] Chen Y Feng R Yang X Dai J Huang M Ji X Yogurt improves insulin resistance and liver fat in obese women with nonalcoholic fatty liver disease and metabolic syndrome: a randomized controlled trial. Am J Clin Nutr. 2019;109:1611-9. 10.1093/ajcn/nqy358 31136662

[32] Daniel N Nachbar RT Tran TTT Ouellette A Varin TV Cotillard A Gut microbiota and fermentation-derived branched chain hydroxy acids mediate health benefits of yogurt consumption in obese mice. Nat Commun. 2022;13:1343. 10.1038/s41467-022-29005-0 35292630 PMC8924213

[33] Hirahatake KM Astrup A Hill JO Slavin JL Allison DB Maki KC Potential Cardiometabolic Health Benefits of Full-Fat Dairy: The Evidence Base. Adv Nutr. 2020;11:533–47. 10.1093/advances/nmz132 31904812 PMC7231591

[34] Gao X Jia HY Chen GC Li CY Hao M Yogurt Intake Reduces All-Cause and Cardiovascular Disease Mortality: A Meta-Analysis of Eight Prospective Cohort Studies. Chin J Integr Med. 2020;26:462–8. 10.1007/s11655-020-3085-8 31970674

[35] Lu W Chen H Niu Y Wu H Xia D Wu Y Dairy products intake and cancer mortality risk: a meta-analysis of 11 population-based cohort studies. Nutr J. 2016;15:91. 10.1186/s12937-016-0210-9 27765039 PMC5073921

[36] Tamura T Wakai K Kato Y Tamada Y Kubo Y Okada R Dietary Carbohydrate and Fat Intakes and Risk of Mortality in the Japanese Population: the Japan Multi-Institutional Collaborative Cohort Study. J Nutr. 2023;153:2352–68. 10.1016/j.tjnut.2023.05.027 37271417

[37] Nakanishi A Homma E Osaki T Sho R Souri M Sato H Association between milk and yogurt intake and mortality: a community-based cohort study (Yamagata study). BMC Nutr. 2021;7:33. 10.1186/s40795-021-00435-1 34256873 PMC8278744

