# Supplementary material for: Moderate full-fat and low-fat yoghurt consumption correlates with reduced mortality risk: a large-scale prospective analysis
Source: J Glob Health. 2025 Jan 17;15:04014. doi: 10.7189/jogh.15.04014 (PMC11737816; doi:10.7189/jogh.15.04014)
Supplement: Online Supplementary Document [file jogh-15-04014-s001.pdf]

Table S1. Cox proportional hazard model of the association between low-fat and full-fat yogurt consumption and all-cause, cancer, CVD mortality

Outcome	Nondrinkers	Low-fat yogurt	Full-fat yogurt	Both types
**All causes**				
Events, n (%)	5391 (5.4)	2568 (4.7)	553 (4.3)	890 (4.7)
Model				
0	1 (reference)	0.87 (0.83-0.91)	0.77 (0.71-0.85)	0.86 (0.80-0.92)
1	1 (reference)	0.86 (0.82-0.90)	0.81 (0.74-0.88)	0.82 (0.77-0.88)
2	1 (reference)	0.90 (0.86-0.94)	0.89 (0.81-0.97)	0.88 (0.82-0.95)
3	1 (reference)	0.93 (0.88-0.97)	0.91 (0.83-0.99)	0.91 (0.85-0.98)
**Cancer cause**				
Events, n (%)	2838 (2.8)	1432 (2.6)	314 (2.4)	489 (2.6)
Model				
0	1 (reference)	0.92 (0.87-0.98)	0.84 (0.75-0.94)	0.90 (0.81-0.99)
1	1 (reference)	0.89 (0.83-0.95)	0.86 (0.76-0.96)	0.84 (0.76-0.92)
2	1 (reference)	0.93 (0.87-0.99)	0.92 (0.82-1.04)	0.89 (0.81-0.98)
3	1 (reference)	0.95 (0.89-1.02)	0.94 (0.84-1.06)	0.91 (0.83-1.01)
**CVD cause**				
Events, n (%)	981 (1.0)	466 (0.9)	92 (0.7)	148 (0.8)
Model				
0	1 (reference)	0.87 (0.78-0.97)	0.71 (0.57-0.88)	0.78 (0.66-0.93)

Outcome	Nondrinkers	Low-fat yogurt	Full-fat yogurt	Both types
**All causes**				
Events, n (%)	5391 (5.4)	2568 (4.7)	553 (4.3)	890 (4.7)
1	1 (reference)	0.90 (0.80-1.00)	0.75 (0.60-0.93)	0.78 (0.66-0.93)
2	1 (reference)	0.95 (0.85-1.06)	0.87 (0.70-1.08)	0.86 (0.73-1.03)
3	1 (reference)	0.97 (0.86-1.08)	0.88 (0.71-1.09)	0.89 (0.75-1.06)

Model 0: unadjusted

Model 1: adjusted for sex, age, TDI, ethnicity, and education.

Model 2: model 1 also adjusted for smoking status, BMI, physical activity level, Alcohol drinking, high cholesterol, hypertension, diabetes, long-standing illness, cholesterol-lowering drug use, blood pressure drug use, and insulin drug use.

Model 3: model 2 also adjusted for vitamin and mineral supplement use (yes or no [vitamin A, B, C, D, or E; folic acid; or multivitamins or minerals]), tea consumption, SSBs, ASBs, and intake of total energy, fat, total sugar, fresh fruit, vegetables, red meat, processed meat, water, coffee, milk pudding, milk chocolate, cheese, and milk.

N number, HR hazard ratio, CI confidence interval

Table S2. Dose-response associations of low-fat and full-fat yogurt consumption with all-cause, cancer, and CVD mortality after excluding participants with missing covariates

Outcome	Nondrinkers	Yogurt drinkers		
		<50 g/day	>50, <100 g/day	>100 g/day
**Low-fat yogurt**				
**All-cause**				
Basic model	1(reference)	0.78 (0.72-0.86); P<0.001	0.92 (0.82-1.03); P: 0.144	0.90 (0.77-1.05); P: 0.174
Multivariable model	1(reference)	0.89 (0.81-0.97); P: 0.013	1.01 (0.90-1.13); P: 0.897	0.97 (0.83-1.13); P: 0.673
**Cancer cause**				
Basic model	1(reference)	0.81 (0.71-0.91); P<0.001	0.93 (0.79-1.08); P: 0.320	0.94 (0.76-1.15); P: 0.549
Multivariable model	1(reference)	0.90 (0.79-1.02); P: 0.094	1.00 (0.86-1.17); P: 0.960	1.00 (0.82-1.24); P: 0.971
**CVD cause**				
Basic model	1(reference)	0.75(0.60-0.94); P: 0.011	0.81(0.61-1.07); P: 0.141	0.81(0.55-1.19); P: 0.275
Multivariable model	1(reference)	0.88(0.70-1.10); P: 0.269	0.90(0.68-1.20); P: 0.487	0.88(0.60-1.30); P: 0.521
**Full-fat yogurt**				
**All-cause**				
Basic model	1(reference)	0.81 (0.75-0.88); P<0.001	0.85 (0.79-0.92); P<0.001	0.92 (0.85-0.98); P: 0.017
Multivariable model	1(reference)	0.88 (0.81-0.96); P: 0.004	0.92 (0.85-1.00); P: 0.039	0.97 (0.90-1.04); P: 0.366
**Cancer cause**				
Basic model	1(reference)	0.83(0.75-0.93); P: 0.001	0.89(0.81-0.98); P: 0.024	0.89(0.81-0.98); P: 0.021
Multivariable model	1(reference)	0.90(0.81-1.01); P: 0.065	0.96(0.87-1.07); P: 0.474	0.95(0.86-1.05); P: 0.312
**CVD cause**				
Basic model	1(reference)	0.78(0.64-0.95); P: 0.015	0.79(0.66-0.95); P: 0.013	0.88(0.74-1.05); P: 0.163
Multivariable model	1(reference)	0.88(0.72-1.07); P: 0.192	0.87(0.72-1.05); P: 0.137	0.91(0.76-1.08); P: 0.282

Basic model: adjusted for sex, age, TDI, ethnicity, and education.

Multivariable model: adjusted for sex, age, TDI, ethnicity, education, smoking status, BMI, physical activity level, Alcohol drinking, high cholesterol, hypertension, diabetes, long-standing illness, cholesterol-lowering drug use, blood pressure drug use, insulin drug use, vitamin and mineral supplement use (yes or no [vitamin A, B, C, D, or E; folic acid; or multivitamins or minerals]), tea consumption, SSBs, ASBs, and intake of total energy, fat, total sugar, fresh fruit, vegetables, red meat, processed meat, water, coffee, milk pudding, milk chocolate, cheese, and milk.

Table S3. Dose-response associations of low-fat and full-fat yogurt consumption with all-cause, cancer, and CVD mortality after excluding participants who use vitamins or minerals.

Outcome	Nondrinkers	Yogurt drinkers		
		**<50 g/day**	**50-100 g/day**	**>100 g/day**
**Low-fat yogurt**				
**All-cause**				
Basic model	1 (reference)	0.75 (0.69-0.83); P<0.001	0.93 (0.83-1.05); P: 0.238	0.85 (0.72-1.00); P: 0.053
Multivariable model	1 (reference)	0.85 (0.78-0.94); P: 0.002	1.03 (0.92-1.16); P: 0.565	0.95 (0.81-1.12); P: 0.525
**Cancer cause**				
Basic model	1 (reference)	0.83 (0.74-0.94); P: 0.004	0.89 (0.76-1.04); P: 0.152	0.97 (0.78-1.19); P: 0.737
Multivariable model	1 (reference)	0.92 (0.81-1.04); P: 0.183	0.97 (0.82-1.14); P: 0.705	1.06 (0.86-1.31); P: 0.571
**CVD cause**				
Basic model	1(reference)	0.71 (0.56-0.90); P: 0.004	0.97 (0.74-1.27); P: 0.850	0.78 (0.52-1.16); P: 0.226
Multivariable model	1 (reference)	0.84 (0.66-1.06); P: 0.137	1.11 (0.85-1.46); P: 0.451	0.89 (0.59-1.33); P: 0.565
**Full-fat yogurt**				
**All-cause**				
Basic model	1 (reference)	0.79 (0.72-0.86); P<0.001	0.88 (0.82-0.95); P: 0.001	0.88 (0.81-0.95); P<0.001
Multivariable model	1 (reference)	0.86 (0.79-0.94); P<0.001	0.96 (0.89-1.04); P: 0.296	0.93 (0.86-1.01); P: 0.071
**Cancer cause**				
Basic model	1 (reference)	0.86 (0.77-0.96); P: 0.007	0.92 (0.83-1.02); P: 0.108	0.87 (0.79-0.97); P: 0.009
Multivariable model	1 (reference)	0.92 (0.82-1.03); P: 0.139	0.99 (0.89-1.10); P: 0.882	0.94 (0.84-1.04); P: 0.215
**CVD cause**				
Basic model	1 (reference)	0.72 (0.58-0.89); P: 0.002	0.91 (0.76-1.09); P: 0.325	0.92 (0.77-1.09); P: 0.337
Multivariable model	1 (reference)	0.81 (0.66-1.01); P: 0.057	1.01 (0.84-1.22); P: 0.887	0.96 (0.81-1.15); P: 0.692

Basic model: adjusted for sex, age, TDI, ethnicity and education.

Multivariable model: adjusted for sex, age, TDI, ethnicity, education, smoking status, BMI, physical activity level, Alcohol drinking, high cholesterol, hypertension, diabetes, long-standing illness, cholesterol-lowering drug use, blood pressure drug use, insulin drug use, vitamin and mineral supplement use (yes or no [vitamin A, B, C, D, or E; folic acid; or multivitamins or minerals]), tea consumption, SSBs, ASBs, and intake of total energy, fat, total sugar, fresh fruit, vegetables, red meat, processed meat, water, coffee, milk pudding, milk chocolate, cheese, and milk.

Table S4. Dose-response associations of low-fat and full-fat yogurt consumption with all-cause, cancer, and CVD mortality after excluding participants who reported an outcome event during the first 2 years of follow-up

Outcome	Nondrinkers	Yogurt drinkers		
		<50 g/day	50--100 g/day	>100 g/day
**Low-fat yogurt**				
**All-cause**				
Basic model	1 (reference)	0.78 (0.72-0.84); P<0.001	0.91 (0.82-1.00); P: 0.046	0.90 (0.79-1.02); P: 0.109
Multivariable model	1 (reference)	0.88 (0.81-0.95); P: 0.001	1.01 (0.91-1.11); P: 0.890	0.98 (0.86-1.12); P: 0.820
**Cancer cause**				
Basic model	1 (reference)	0.82 (0.74-0.91); P<0.001	0.88 (0.77-1.01); P: 0.061	0.96 (0.81-1.14); P: 0.627
Multivariable model	1 (reference)	0.91 (0.82-1.01); P: 0.086	0.96 (0.84-1.10); P: 0.557	1.04 (0.87-1.24); P: 0.654
**CVD cause**				
Basic model	1 (reference)	0.71 (0.59-0.86); P<0.001	0.94 (0.75-1.18); P: 0.622	0.81 (0.59-1.12); P: 0.203
Multivariable model	1 (reference)	0.84 (0.69-1.02); P: 0.077	1.08 (0.86-1.36); P: 0.489	0.91 (0.65-1.26); P: 0.556
**Full-fat yogurt**				
**All-cause**				
Basic model	1(reference)	0.81 (0.75-0.87); P<0.001	0.86 (0.81-0.92); P<0.001	0.90 (0.84-0.95); P<0.001
Multivariable model	1(reference)	0.88 (0.82-0.95); P<0.001	0.93(0.87-1.00); P: 0.038	0.95 (0.89-1.01); P: 0.113
**Cancer cause**				
Basic model	1 (reference)	0.86 (0.78-0.94); P: 0.001	0.89 (0.82-0.97); P: 0.010	0.89 (0.82-0.97); P: 0.005
Multivariable model	1 (reference)	0.93 (0.84-1.02); P: 0.119	0.97 (0.89-1.05); P: 0.445	0.95 (0.87-1.03); P: 0.227
**CVD cause**				
Basic model	1 (reference)	0.80 (0.68-0.94); P: 0.008	0.88 (0.76-1.02); P: 0.098	0.94 (0.81-1.08); P: 0.377
Multivariable model	1 (reference)	0.90 (0.76-1.06); P: 0.206	0.97 (0.83-1.13); P: 0.729	0.98 (0.85-1.14); P: 0.796

Basic model: adjusted for sex, age, TDI, ethnicity, and education.

Multivariable model: adjusted for sex, age, TDI, ethnicity, education, smoking status, BMI, physical activity level, Alcohol drinking, high cholesterol, hypertension, diabetes, long-standing illness, cholesterol-lowering drug use, blood pressure drug use, insulin drug use, vitamin and mineral supplement use (yes or no [vitamin A, B, C, D, or E; folic acid; or multivitamins or minerals]), tea consumption, SSBs, ASBs, and intake of total energy, fat, total sugar, fresh fruit, vegetables, red meat, processed meat, water, coffee, milk pudding, milk chocolate, cheese, and milk.

Table S5. Dose-response associations of low-fat and full-fat yogurt consumption with all-cause, cancer, and CVD mortality after adjusting for environmental factors

Outcome	Nondrinkers	Yogurt drinkers		
		**<50 g/day**	**50-100 g/day**	**>100 g/day**
**Low-fat yogurt**				
**All-cause**				
Basic model	1 (reference)	0.76 (0.71-0.82); P<0.001	0.90 (0.82-0.99); P: 0.029	0.89 (0.79-1.02); P: 0.088
Multivariable model	1 (reference)	0.87(0.80-0.94); P<0.001	1.00 (0.91-1.10); P: 0.980	0.98 (0.86-1.11); P: 0.734
**Cancer cause**				
Basic model	1(reference)	0.81 (0.73-0.90); P<0.001	0.86 (0.76-0.99); P: 0.031	0.95 (0.80-1.13); P: 0.562
Multivariable model	1(reference)	0.90 (0.81-1.00); P: 0.050	0.94 (0.83-1.08); P: 0.390	1.03 (0.87-1.22); P: 0.740
**CVD cause**				
Basic model	1(reference)	0.69 (0.57-0.84); P<0.001	0.93 (0.74-1.16); P: 0.515	0.79 (0.57-1.09); P: 0.145
Multivariable model	1(reference)	0.82 (0.68-1.00); P: 0.050	1.07 (0.85-1.34); P: 0.577	0.87 (0.63-1.21); P: 0.421
**Full-fat yogurt**				
**All-cause**				
Basic model	1 (reference)	0.79 (0.74-0.85); P<0.001	0.85 (0.80-0.91); P<0.001	0.90 (0.84-0.95); P<0.001
Multivariable model	1 (reference)	0.87 (0.81-0.94); P<0.001	0.93 (0.87-0.99); P: 0.019	0.95 (0.89-1.01); P: 0.105
**Cancer cause**				
Basic model	1 (reference)	0.85 (0.77-0.93); P<0.001	0.88 (0.81-0.96); P: 0.004	0.89 (0.82-0.96); P: 0.004
Multivariable model	1 (reference)	0.92 (0.83-1.01); P: 0.068	0.96 (0.88-1.04); P: 0.316	0.95 (0.87-1.03); P: 0.205
**CVD cause**				
Basic model	1 (reference)	0.78 (0.66-0.92); P: 0.003	0.87 (0.75-1.01); P: 0.061	0.93 (0.81-1.07); P: 0.329
Multivariable model	1 (reference)	0.88 (0.74-1.04); P: 0.140	0.96 (0.83-1.12); P: 0.642	0.98 (0.84-1.13); P: 0.740

Basic model: adjusted for sex, age, TDI, ethnicity, and education.

Multivariable model: adjusted for sex, age, TDI, ethnicity, education, smoking status, BMI, physical activity level, Alcohol drinking, high cholesterol, hypertension, diabetes, long-standing illness, cholesterol-lowering drug use, blood pressure drug use, insulin drug use, vitamin and mineral supplement use (yes or no [vitamin A, B, C, D, or E; folic acid; or multivitamins or minerals]), tea consumption, SSBs, ASBs, and intake of total energy, fat, total sugar, fresh fruit, vegetables, red meat, processed meat, water, coffee, milk pudding, milk chocolate, cheese, milk, PM2.5, PM2.5-10, PM10, NOx, inverse distance to major road, green space percentage, average sound level.

Table S6. Dose-response associations of c low-fat and full-fat yogurt consumption with all-cause, cancer, and CVD mortality after excluding participants reported less than two measurements of yogurt consumption.

Outcome	Nondrinkers	Yogurt drinkers		
		<50 g/day	50-100 g/day	>100 g/day
**Low-fat yogurt**				
**All-cause**				
Basic model	1 (reference)	0.83 (0.76-0.90); P<0.001	0.97 (0.86-1.10); P: 0.655	0.86 (0.68-1.08); P: 0.196
Multivariable model	1 (reference)	0.90 (0.83-0.98); P: 0.019	1.07 (0.95-1.21); P: 0.282	0.95 (0.75-1.20); P: 0.677
**Cancer cause**				
Basic model	1 (reference)	0.85 (0.76-0.96); P: 0.006	0.95 (0.80-1.12); P: 0.519	0.89 (0.65-1.21); P: 0.445
Multivariable model	1 (reference)	0.92 (0.82-1.03); P: 0.168	1.02 (0.87-1.21); P: 0.794	0.97 (0.71-1.32); P: 0.848
**CVD cause**				
Basic model	1 (reference)	0.76 (0.61-0.93); P: 0.008	0.94 (0.70-1.26); P: 0.681	0.78 (0.44-1.38); P: 0.396
Multivariable model	1 (reference)	0.83 (0.68-1.03); P: 0.092	1.05(0.79-1.41); P: 0.729	0.88 (0.50-1.57); P: 0.671
**Full-fat yogurt**				
**All-cause**				
Basic model	1 (reference)	0.86 (0.80-0.93); P<0.001	0.91 (0.85-0.98); P: 0.019	0.90 (0.81-0.98); P: 0.023
Multivariable model	1 (reference)	0.91 (0.85-0.99); P: 0.023	0.97 (0.90-1.05); P: 0.425	0.95 (0.86-1.05); P: 0.326
**Cancer cause**				
Basic model	1 (reference)	0.90 (0.81-0.99); P: 0.037	0.96 (0.86-1.06); P: 0.377	0.95 (0.84-1.08); P: 0.430
Multivariable model	1 (reference)	0.95 (0.86-1.05); P: 0.314	1.02 (0.92-1.12); P: 0.765	1.02 (0.90-1.15); P: 0.798
**CVD cause**				
Basic model	1 (reference)	0.84 (0.70-1.01); P: 0.067	0.91 (0.76-1.09); P: 0.315	0.71 (0.55-0.92); P: 0.008

Multivariable model	1 (reference)	0.89 (0.74-1.07); P: 0.209	0.96 (0.80-1.15); P: 0.671	0.75 (0.58-0.97); P: 0.029

Basic model: adjusted for sex, age, TDI, ethnicity, and education.

Multivariable model: adjusted for sex, age, TDI, ethnicity, education, smoking status, BMI, physical activity level, Alcohol drinking, high cholesterol, hypertension, diabetes, long-standing illness, cholesterol-lowering drug use, blood pressure drug use, insulin drug use, vitamin and mineral supplement use (yes or no [vitamin A, B, C, D, or E; folic acid; or multivitamins or minerals]), tea consumption, SSBs, ASBs, and intake of total energy, fat, total sugar, fresh fruit, vegetables, red meat, processed meat, water, coffee, milk pudding, milk chocolate, cheese, and milk.

Table S7. Dose-response associations of low-fat and full-fat yogurt consumption with all-cause, cancer, and CVD mortality after removing participants with high within-person variability (standard difference greater than 50g per day) across the five assessments

Outcome	Nondrinkers	Yogurt drinkers		
		**<50 g/day**	**50-100 g/day**	**>100 g/day**
**Low-fat yogurt**				
**All-cause**				
Basic model	1(reference)	0.83 (0.75-0.91); P<0.001	0.75 (0.54-1.05); P: 0.091	0.91 (0.68-1.23); P: 0.552
Multivariable model	1 (reference)	0.90 (0.81-0.99); P: 0.039	0.86 (0.62-1.20); P: 0.382	1.03 (0.76-1.38); P: 0.858
**Cancer cause**				
Basic model	1 (reference)	0.86 (0.75-0.98); P: 0.022	0.79 (0.51-1.21); P: 0.277	0.93 (0.63-1.38); P: 0.715
Multivariable model	1 (reference)	0.93 (0.81-1.06); P: 0.265	0.88 (0.57-1.36); P: 0.572	1.03 (0.69-1.53); P: 0.892
**CVD cause**				
Basic model	1 (reference)	0.69 (0.54-0.89); P: 0.005	0.36 (0.12-1.12); P: 0.078	1.01 (0.52-1.95); P: 0.986
Multivariable model	1 (reference)	0.76 (0.59-0.98); P: 0.037	0.44 (0.14-1.38); P: 0.158	1.19 (0.61-2.31); P: 0.609
**Full-fat yogurt**				
**All-cause**				
Basic model	1 (reference)	0.87 (0.78-0.96); P: 0.006	0.82 (0.69-0.97); P: 0.022	0.95 (0.85-1.07); P: 0.428
Multivariable model	1 (reference)	0.95 (0.86-1.05); P: 0.321	0.92 (0.78-1.10); P: 0.360	1.02 (0.91-1.15); P: 0.721
**Cancer cause**				
Basic model	1 (reference)	0.87 (0.76-1.00); P: 0.052	0.78 (0.62-0.99); P: 0.041	1.04 (0.89-1.21); P: 0.641
Multivariable model	1 (reference)	0.95 (0.83-1.09); P: 0.474	0.88 (0.69-1.12); P: 0.299	1.12 (0.96-1.31); P: 0.136
**CVD cause**				
Basic model	1 (reference)	0.84 (0.66-1.07); P: 0.162	0.82 (0.55-1.23); P: 0.330	0.73 (0.54-1.00); P: 0.050

Multivariable model	1 (reference)	0.93 (0.73-1.19); P: 0.559	0.94 (0.63-1.42); P: 0.776	0.77 (0.56-1.05); P: 0.104

Basic model: adjusted for sex, age, TDI, ethnicity, and education.

Multivariable model: adjusted for sex, age, TDI, ethnicity, education, smoking status, BMI, physical activity level, Alcohol drinking, high cholesterol, hypertension, diabetes, long-standing illness, cholesterol-lowering drug use, blood pressure drug use, insulin drug use, vitamin and mineral supplement use (yes or no [vitamin A, B, C, D, or E; folic acid; or multivitamins or minerals]), tea consumption, SSBs, ASBs, and intake of total energy, fat, total sugar, fresh fruit, vegetables, red meat, processed meat, water, coffee, milk pudding, milk chocolate, cheese, and milk.
